# Molecular detection of dengue virus in patients suspected of Ebola virus disease in Ghana

**DOI:** 10.1371/journal.pone.0208907

**Published:** 2018-12-19

**Authors:** Joseph Humphrey Kofi Bonney, Takaya Hayashi, Samuel Dadzie, Esinam Agbosu, Deborah Pratt, Stephen Nyarko, Franklin Asiedu-Bekoe, Eiji Ido, Badu Sarkodie, Nobuo Ohta, Shoji Yamaoka

**Affiliations:** 1 Virology Department, Noguchi Memorial Institute of Medical Research, University of Ghana, Legon, Ghana; 2 Tokyo Medical and Dental University, Tokyo, Japan; 3 Disease Surveillance Department, Ghana Health Service, Accra, Ghana; 4 Public Health Division, Ghana Health Service, Accra, Ghana; CEA, FRANCE

## Abstract

Dengue fever is known to be one of the most common arthropod-borne viral infectious diseases of public health importance. The disease is now endemic in more than 100 countries in Africa, the Americas, the Eastern Mediterranean, Southeast Asia and the Western Pacific with an estimated two fifths of the world's population being at risk. The notable endemic viral hemorrhagic fevers (VHFs) found in West Africa, including yellow fever, Lassa fever, Rift Valley fever, dengue fever and until recently Ebola have been responsible for most outbreaks with fatal consequences. These VHFs usually produce unclear acute febrile illness, especially in the acute phase of infection. In this study we detected the presence of 2 different serotypes (DENV-2 and DENV-3) of Dengue virus in 4 sera of 150 patients clinically suspected of Ebola virus disease during the Ebola Virus Disease (EVD) outbreak in West Africa with the use of serological and molecular test assays. Sequence data was successfully generated for DENV-3 and phylogenetic analysis of the envelope gene showed that the DENV-3 sequences had close homology with DENV-3 sequences from Senegal and India. This study documents molecular evidence of an indigenous Dengue fever viral infection in Ghana and therefore necessitates the need to have an efficient surveillance system to rapidly detect and control the dissemination of the different serotypes in the population which has the potential to cause outbreaks of dengue hemorrhagic fevers.

## Introduction

Viral hemorrhagic fevers (VHFs), which refer to a group of illnesses that are caused by viruses within a distinct group of families, are well known for their characteristic overall vascular system damage and the impairment of the body’s ability to regulate itself [1]. Symptoms presented by VHF-related illnesses are often accompanied by hemorrhage which in itself is rarely life-threatening [1]. While some types of hemorrhagic fever viruses cause relatively mild illness, many of these viruses cause severe fatal disease. The occurrence of outbreaks caused by these viruses cannot be certainly projected However, in recent times, the world has come to terms with the devastating impact of mortality and morbidity unleashed by two viral agents within these distinct viral groups–Dengue virus and Ebola virus. While the mortality rate of the latter is known to be a little over 90% [2] in some instances, it is rather the morbidity rate in the former that causes extensive concern as the World Health Organization (WHO) indicated a 30-fold increase in cases since 1960 [3].

DF which is considered the most important *Aedes* mosquito-borne viral disease to infect humans is caused by one of four closely related serotypes of Dengue virus (DENV) [4]. The four serotypes, DENV serotypes 1–4, have caused large urban outbreaks, particularly once the co-circulation of different serotypes is observed or when a new serotype is presented [5–7]. The virus belongs to the genus Flavivirus within the *Flaviviridae* family [8]. It is endemic and known as a common illness throughout Southeast Asia and much of the Americas [8]. A study that used a cartographic approach provided an annual global estimate of 390 million DENV infections of which 67 to 136 million showed at any level of disease severity [9]. In this same study 70% of the global estimated infections were predicted to have occurred in Asia and 16% in Africa [9,10]. It was however noted with interest the large estimated burden of Dengue in Africa from this study as it was not until recently that the often-recognized disease outside Africa has assumed higher levels of risk in Africa [9,10].

The widespread epidemic of Ebola virus disease (EVD) in three West African countries, Guinea, Sierra Leone and Liberia, between 2013 and 2015 caused significant mortality, with reported case fatality rates of up to 72% [11] and specifically 57–59% among hospitalized patients [11]. Many West African countries including Ghana that are near to these three most affected countries were deemed at high risk of EVD importation. As a response to the outbreak in these neighboring countries, a national surveillance system was set up in Ghana as part of an EVD preparedness and response plan to detect and rapidly respond to cases and rumors of cases. Clinical specimens of serum/plasma from patients suspected of EVD from health facilities across the country were submitted to the Noguchi Memorial Institute for Medical Research (NMIMR), which was designated for laboratory investigation of these suspected cases.

To establish a definitive diagnosis for these suspected cases of EVD, a testing algorithm that involved investigating for the other known endemic VHFs in the sub-region was adopted. Thus, laboratory investigations were not only performed for Ebola and Marburg but the other endemic hemorrhagic fever viruses including Yellow fever, Lassa fever West Nile and DF. While a definitive viral diagnosis could not be established for the majority of the suspected cases, four were found to be positive for dengue virus with our testing algorithm. The import of this approach was reinforced when analysis of the case investigation forms that accompanied these suspected specimens suggested only 10% met the WHO standard case definition for an EVD [12]. From the foregoing, we sought to detect viral agents of hemorrhagic fevers likely to be endemic in the country from the suspected clinical specimens submitted for laboratory investigation of EVD. We intend to present the molecular indication of a native Dengue viral infection in Ghana.

## Materials and methods

### Clinical specimens

The clinical specimens of sera and plasma used in this study were archived specimens stored at Noguchi Memorial Institute for Medical Research (NMIMR). These samples had been collected between 2014 and 2016, during the West African EVD outbreak. Eligible patients were examined by trained health staff and diagnosed on suspicion of EVD illness based on the routine surveillance case definition of the standard WHO case definitions for Ebola and Marburg virus diseases [12]. During that period, they were tested for EVD and other VHFs including Marburg, Lassa fever, yellow fever and West Nile by reverse transcription polymerase chain reaction (RT-PCR). Residual specimens were stored at -80°C. Thus, the batch of specimens used in this work had previously been tested for EVD, Marburg, Lassa fever and yellow fever but were yet to be tested for other flaviviruses.

Ethical approval was obtained from the Noguchi Memorial Institute for Medical Research Institutional Review Board (NMIMR-IRB) and the Ghana Health Service for this research work (study number: #033/15-16). All analyzed patients’ clinical specimens used in this study were anonymized prior to access by the authors.

### Serological assays

The 150 residual clinical specimens were individually tested with serological assays for the presence of antibodies to DENV. Test assays were performed in a 96-well ELISA format using commercially available kit and test procedure followed in accordance with manufacturer’s instructions: Human Anti-DENV IgG/IgM ELISA kit (Abcam, Cambridge, UK). Anti-DENV IgM positives were tested with a lateral flow immunochromatographic strip (Dengue NS1 Ag STRIP; Bio-Rad, Marnes-la-Coquette, France).

### Nucleic acid extraction and purification

Viral RNA was extracted from 140 μL of clinical specimens that were anti-DENV IgM positive with the QIAmp viral RNA kit (Qiagen, Hilden, Germany) according to the manufacturer’s instructions. The extracted and purified nucleic acid was eluted from the spin columns with 60 μL of RNase/DNase-free elution buffer provided with the kit.

### Nucleic acid amplification assays

To amplify the nucleic acid and detect the genomic sequence of interest, several amplification assays were deployed. An initial test run was carried out with a TaqMan-based real time reverse transcription-polymerase chain reaction (rRT-PCR) assay developed by Johnson et al. [13] to detect and type the four DENV serotypes. In a 25μl reaction mixtures with the Applied Biosystems 7300 RT-PCR system (Life Technologies, Grand Island, NY, USA), volumes each of 5μl of extracted and purified RNA from the clinical sera or plasma were combined with 10 pmol each of Dengue serotype specific primer and probe sets and targets. Each reaction mixture contained a single Dengue serotype primer pair and probe–making four separate reactions for the isolated and purified RNAs for each clinical specimen. The reagent master mix was prepared according to the number of reactions required for each test run using the AgPath-ID One-Step RT-PCR kit (#AM1005, Thermo Fisher Scientific, NY, USA). Then a final addition of 5μl of individual RNA to each appropriately labeled well. Included in each test run were ‘no RNA’ or nuclease-free water reagent and Dengue positive RNA control. A presumptive positive for Dengue serotype specific virus was considered for a clinical specimen when the controls met stated requirements and the growth curves crossed the threshold line within 40 cycles with the inverse true for negatively considered clinical specimens for Dengue serotype specific virus.

### Conventional RT-PCR

In all, four conventional RT-PCR assays were performed and, in each test, run a volume of 25 μl with 5 μl nucleic acid extract was used as a template. The reagents, cycle numbers, primer sequences, target regions and amplicon lengths are shown in Table 1. The four conventional RT-PCR assays were performed using the Aeris G-96 well PCR system (Esco Micro Pte Ltd, Singapore). The amplification products were electrophoresed on a 2% Agarose gel (peqlab Biotechnologie, Erlangen, Germany), stained with Ethidium bromide, and viewed under UV light.

**Table 1:** Dengue virus primers and RT-PCR assays used.

Primer Name	Reagents	Cycles	Sequences (5'-3')	Target gene	Genome position/ Amplicon Length		Reference
**Real-time PCR**							
**DENV-1**							
DEN-1 Forward	AgPath-ID One-Step RT-PCR Reagents	45	CAAAAGGAAGTCGTGCAATA	Envelope		8973	[13]
DEN-1 Reverse			CTGAGTGAATTCTCTCTACTGAACC			9084	
DEN-1 Probe			CATGTGGTTGGGAGCACGC			8998	
**DENV-2**							
DEN-2 Forward	AgPath-ID One-Step RT-PCR Reagents	45	CAGGTTATGGCACTGTCACGAT	Envelope		1605	[13]
DEN-2 Reverse			CCATCTGCAGCAACACCATCTC			1583	
DEN-2 Probe			CTCTCCGAGAACAGGCCTCGACTTCAA			1008	
**DENV-3**							
DEN-3 Forward	AgPath-ID One-Step RT-PCR Reagents	45	GGACTGGACACACGCACTCA	Envelope		740	[13]
DEN-3 Reverse			CATGTCTCTACCTTCTCGACTTGTCT			813	
DEN-3 Probe			ACCTGGATGTCGGCTGAAGGAGCTTG			762	
**DENV-4**							
DEN-4 Forward	AgPath-IDOne-Step RT-PCR Reagents	45	TTGTCCTAATGATGCTGGTCG	Envelope		904	[13]
DEN-4 Reverse			TCCACCTGAGACTCCTTCCA			992	
DEN-4 Probe			TTCCTACTCCTACGCATCGCATTCCG			960	
**Conventional PCR**							
DUC	QIAGEN OneStep RT-PCR Kit	45	TCAATATGCTGAAACGCGCGAGAAACCG	Envelope		511	[6]
DUS			TTGCACCAACAGTCAATGTCTTCAGGTTC	Envelope			
NS5F1	QIAGEN OneStep RT-PCR Kit	45	AGYGGAGTRGAAGGRGAAGG	Non-structural 5		917	[46]
NS5F2			AGCATGTCTTCXGTXTCATCCA				
FU1	QIAGEN OneStep RT-PCR Kit	45	TACAACATGATGGGAAAGAGAGAGAA	Non-structural 5		266	[47]
cFD2			GTGTCCCAGCCGGCGGTGTCATCAGC				
FU2	QIAGEN OneStep RT-PCR Kit	45	GCTGATGACACCGCCGGCTGGGACAC	Non-structural 5		845	[47]
cFD3			AGCATGTCTTCCGTGGTCATCCA				

### Sequencing and phylogenetic analysis

The conventional RT-PCR products were sequenced and were assembled using the DNASTAR’s Lasergene sequence analysis software with base calling proofread by graphic examination of the electropherograms. Phylogenetic trees were constructed based on 511 nucleotides of the capsid (pre-membrane) and the 266 nucleotides of the non-structural protein target genomic regions target genes and the analysis included the amplified samples from the conventional RT-PCR as well as GenBank reference sequences of 34 and 37 respectively (Table 2). The reference sequences were selected from representatives of the four DENV serotypes as well as closely related sequences to the query. Phylogenies were inferred by the maximum likelihood method and conducted in MEGA 7 [14]. It was drawn to scale, with branch lengths in the same units as those of the evolutionary distances used.

**Table 2:** A list of reference strains of DENV from the GenBank with their accession numbers, year isolated/detected and the location.

Strain	Serotype	Location	Year		GenBank accession no.
**TB55i**	**3**	**Indonesia**	**2004**	**AY858048**	
**TB16**	**3**	**Indonesia**	**2004**	**AY858047**	
**MKS-WS79b**	**3**	**Indonesia**	**2010**	**KC762693**	
**98902890 DF DV-3**	**3**	**Indonesia**	**1998**	**AB189128**	
**Singapore 8120/95**	**3**	**Singapore**	**2004**	**AY766104**	
**D3/SG/05K4648DK1/2005**	**3**	**Singapore**	**2005**	**EU081225**	
**DENV3-632**	**3**	**Philippines**	**2008**	**KU509279**	
**VIROAF7**	**3**	**Philippines**	**1964**	**KM190937**	
**PhMH-J1-97**	**3**	**Philippines**	**1997**	**AY496879**	
**KDH0010A**	**3**	**Thailand**	**2010**	**HG316483**	
**ThD3 0007 87**	**3**	**Thailand**	**1987**	**AY676353**	
**Pythium**	**3**	**Thailand**	**2014**	**KT424097**	
**DENV-3/WS/BID-V2973/1995**	**3**	**Samoa**	**1995**	**FJ898456**	
**DENV-3/IND/663381/1966**	**3**	**India**	**1966**	**JQ922555**	
**Rajasthan.India/DMRC/Balotra87/2013**	**3**	**India**	**2013**	**KU216208**	
**ND143**	**3**	**India**	**2007**	**FJ644564**	
**UNC3001**	**3**	**Sri Lanka**	**1989**	**JQ411814**	
**DENV-3/LK/BID-V2414/1985**	**3**	**Sri Lanka**	**1985**	**FJ882574**	
**D3/H/IMTSSA-SR/2000/1266**	**3**	**Sri Lanka**	**2000**	**NC_001475**	
**DENV-3/PR/BID-V2116/2001**	**3**	**Puerto Rico**	**2001**	**KF955468**	
**DENV-3/PR/BID-V2102/2000**	**3**	**Puerto Rico**	**2000**	**KF955466**	
**DENV-3/PR/BID-V1728/2006**	**3**	**Puerto Rico**	**2006**	**KF955456**	
**BR74886/02**	**3**	**Brazil**	**2004**	**AY679147**	
**DNV-3/BR/BID-V3609/2007**	**3**	**Brazil**	**2007**	**GU131877**	
**DENV3/BR/D3LIMHO/2006**	**3**	**Brazil**	**2006**	**JN697379**	
**DENV3-3140**	**3**	**Senegal**	**2009**	**KU509282**	
**MKS-0077**	**1**	**Indonesia**	**2007**	**KC762654.1**	
**DENV-1/PH/BID-V2940/2004**	**1**	**Philippines**	**2004**	**GQ868602**	
**16681**	**2**	**Thailand**	**1964**	**NC_001474**	
**7869191/BF/2016**	**2**	**Burkina Faso**	**2016**	**KY627762**	
**UOH_23916**	**4**	**India**	**2015**	**KX845005**	
**BR005AM_2011**	**4**	**Brazil**	**2011**	**KT794007**	

### Statistical analysis

Analysis of quantitative variables were done with non-parametric statistics and statistical tests after data entered in Excel was cleaned. Appropriate measures of central tendency for mean, median, frequency distributions, percentages, and standard deviation were calculated.

### Accession numbers

The sequences of the amplified samples by conventional RT-PCR with two target regions of the capsid and the non-structural 5 gene segments yielded five sequences which were submitted to the DNA Data Bank of Japan (DDBJ) and assigned the accession numbers LC379220—LC379224.

## Results

### ELISA and NS1 serological detection

**Fig 1:**
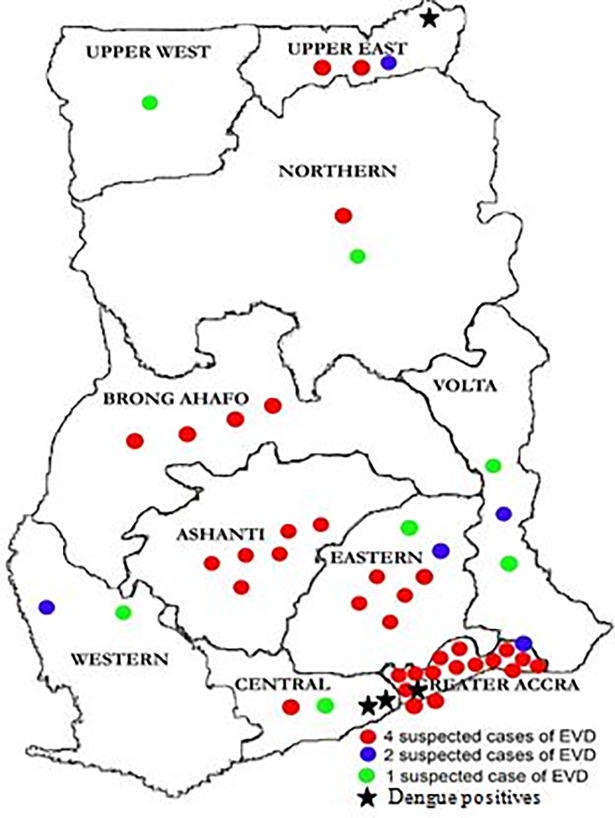
Map of Ghana showing place of origin of samples. Map shows the different parts of the country where samples were collected. The symbol star shows where the four DENV positive samples were taken.

**Fig 2:**
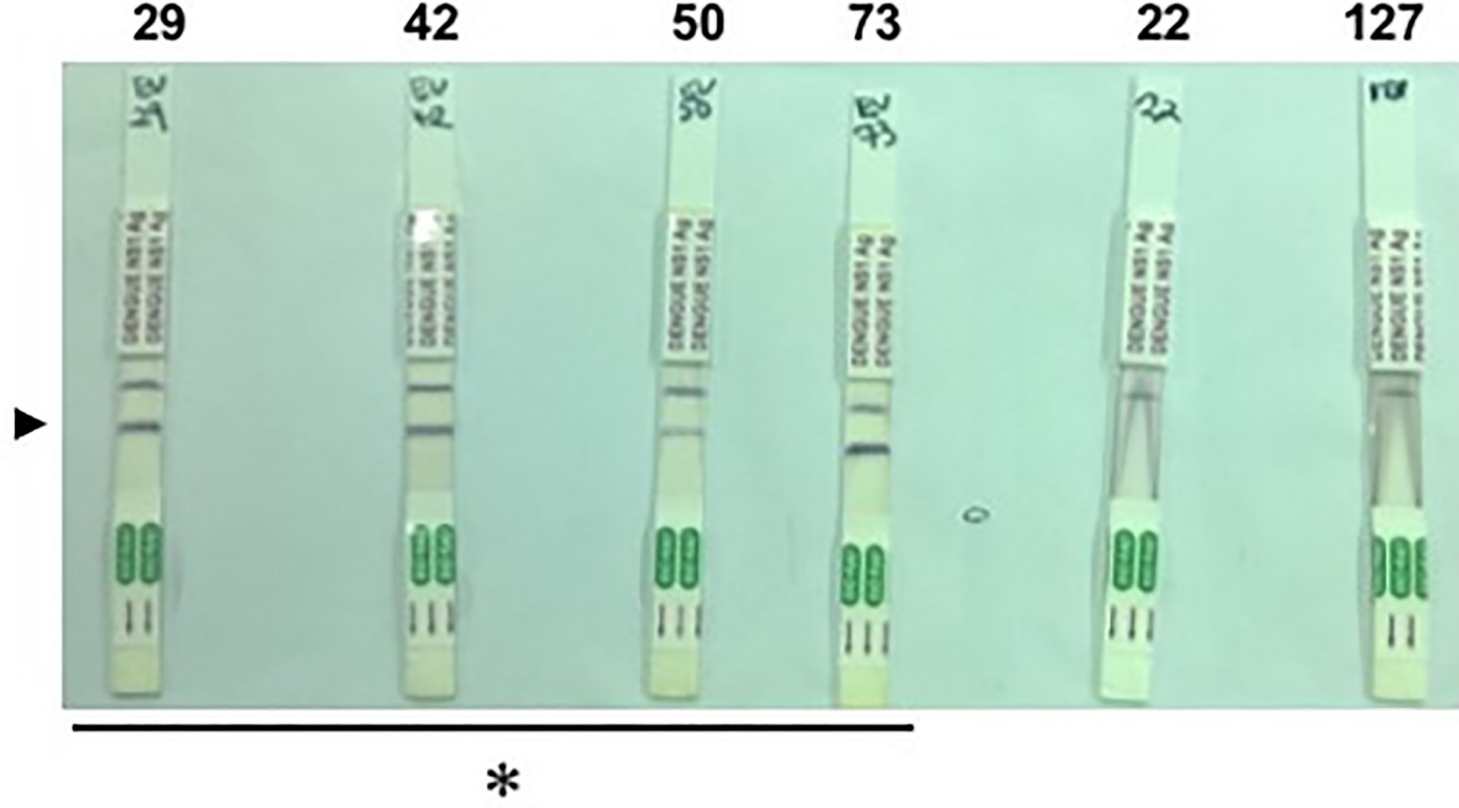
NS1 antigen strips. Immunochromatographic assay for DENV NS1Ag test for real time RT-PCR positive samples (29, 42, 50 and 73). Two samples (22 and 127) that were negative by real time RT-PCR were included for validation. a. Arrowhead: positive band; b. * indicates the four patient samples found to be positive.

The number of archived clinical specimens tested was from 150 patients and collected between the years 2014–2016 from all over the country (Fig 1). Anti-DENV IgM was detected in 32 samples while ant-DENV IgG was detected in 85 samples. Of the 32 anti-DENV IgM positives tested for NS1 Ag, 4 were detected as positive (IDs 29, 42, 50, 73), Table 3, Fig 2.

**Table 3:** Summary of results of testing.

Test Performed	Number tested	Number of Positives (%)
DENV IgG	150	85 (57)
DENV IgM	150	32 (21)
Both DENV IgM and IgG	150	22 (15)
DENV NS1 Antigen	150	4
DENV rRT-PCR	32	4

### PCR assays detection

**Fig 3:**
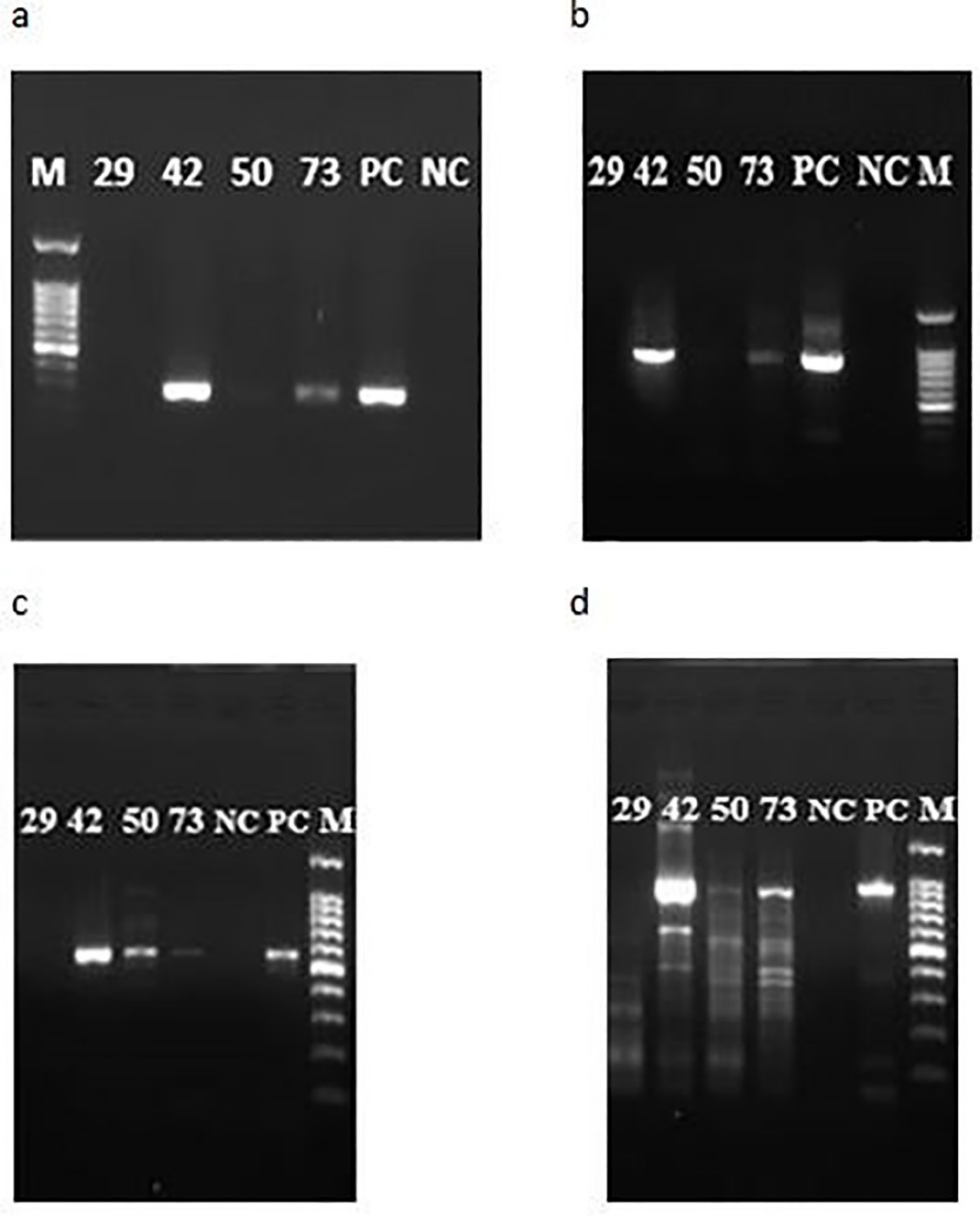
Gel electrophoresis pictures of four positive patient samples by conventional-type RT-PCR using primers indicated. Gel images after amplification using the following primer set: a) FU1 and cFD2- expected size 266bp b) FU2 and cFD3 –expected size 845bp c) DUC and DUS–expected size 511bp d) NS5F1 and NS5R –expected size 917bp NC–Negative control PC–Positive control M–molecular ladder.

All 32 patients’ clinical specimens that were found positive by ELISA for anti-DENV IgM antibodies were analyzed by several PCR assays. Using the TaqMan-based rRT-PCR assay, viral RNA was detected in 4 out of the 32 DENV IgM positive samples. They were characterized as 1 DENV-2 (sample ID 29) and 3 DENV-3 (sample IDs 42, 50 and 73) serotypes. Clear DNA fragments of expected sizes were observed for all but sample 29 when the conventional RT-PCR assays were performed on the 4 positive samples (Fig 3).

### Sequence and phylogenetic analysis

**Fig 4:**
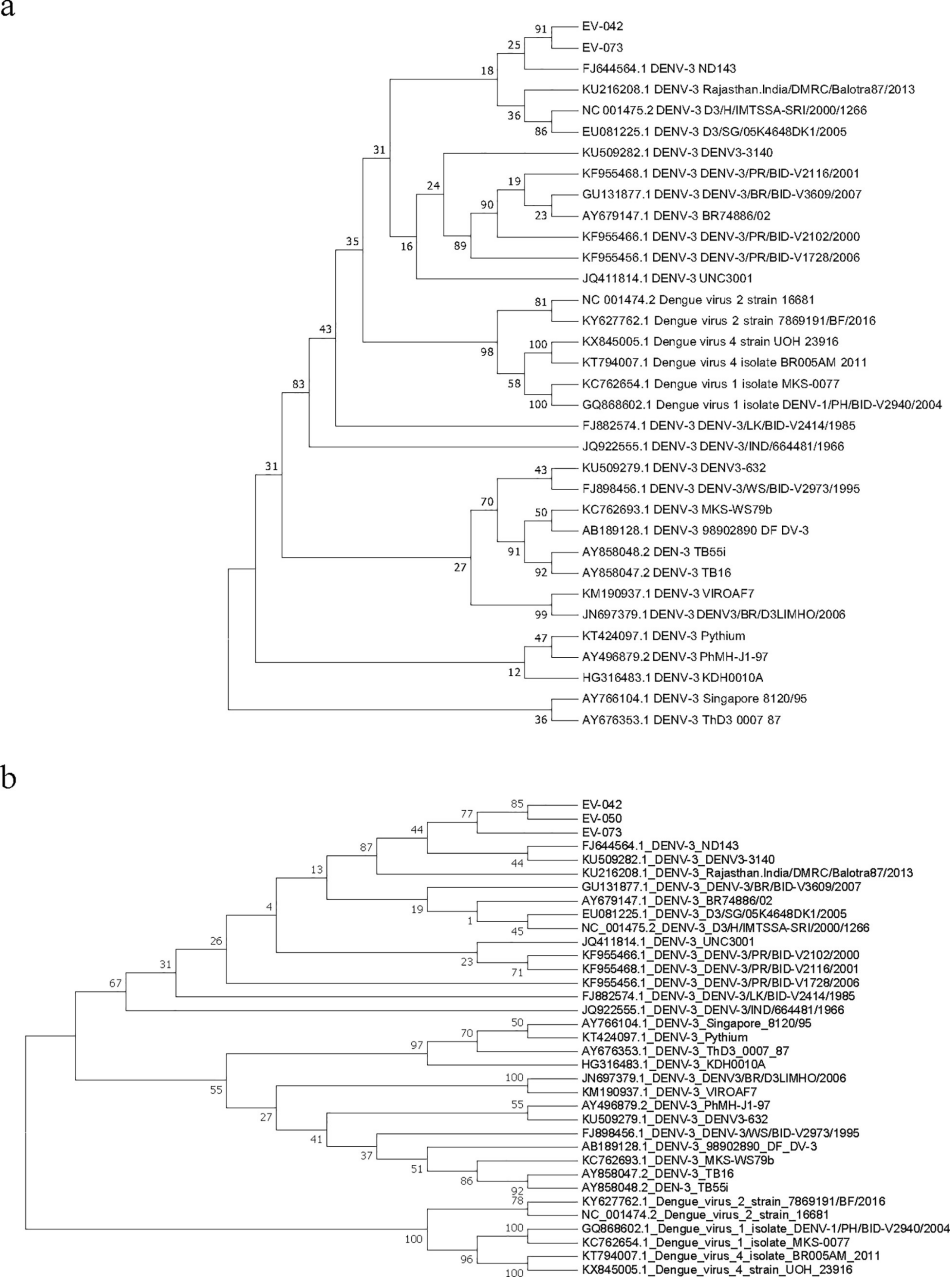
Phylogenetic trees generated based on the non-structural protein and capsid. Phylogenetic trees were drawn using the maximum likelihood method a. Using DUC/DUS sequences b. Using FU1/cFD2 sequences.

To determine the genotypes and evolution of the viruses present in the positive patients’ samples, the amplicons from the conventional RT-PCR run were sequenced and subjected to phylogenetic analysis. The parsimony analysis by maximum likelihood method [15] and MEGA 7 [1] included the three amplified patient’s samples (42, 50 and 73), as well as GenBank reference sequences. In both phylogenetic trees, the patient’s samples were closely related to each other and clustered within DENV serotype 3 and in close homology with sequences from Senegal (KU509282) and India (FJ544564 and KU216208) (Fig 4A and 4B).

### Demographics and clinical details

Of the 4 patients in whom DENV RNA was detected, 2 were males and 2 were females with the age range of 30 to 56years old. Three (IDs 29, 50 and 73) are Ghanaian indigenes residing in the southern part of Ghana (Accra and Kasoa) and one (ID 42) being a Burkinabe who sought medical attention across the border between the two countries in Bawku, a town in the North Eastern part of Ghana. From the clinical characteristics, fever (temperature above 38°C) and muscle/joint pains were the most commonly seen in all patients on presentation. Other observed clinical features included headache, confused or disoriented seizures and unexplained bleeding from the gums and nose (Table 4).

**Table 4:** Demographic and clinical characteristics of dengue positive patients.

Patient ID	Gender	Age (yrs)	Occupation	Nationality	Known Dengue Symptoms	Rare Dengue Symptoms	Outcome
29	Male	56	Trader	Ghanaian	Fever, vomit, nausea and muscle/joint pains	None stated	Alive
42	Male	30	Farmer	Burkinabe	Fever, headache, intense fatigue, general weakness, muscle/joint pains, anorexia/loss of appetite, nose and gum bleeding	Coma, Unconsciousness	Alive
50	Female	33	Trader	Ghanaian	Fever, headache, blood in vomit, muscle/joint pains, abdominal pains, nose and gum bleeding	None stated	Alive
73	Female	35	Housewife	Ghanaian	Fever, vomiting, general weakness, conjunctivitis, headache, muscle/joint pains, nose and gum bleeding, injection site bleeding	Difficulty in breathing, confused and disoriented, seizures, collapse	Dead

## Discussion

A decade ago, the global distribution of the risk of DENV infection and its public health burden were poorly known with little information from Africa [5]. However, recent studies and outbreaks in Burkina Faso and Senegal [16–19] as well as the forces of urbanization and globalization have facilitated the emergence of Dengue and other Arboviruses [5] and increased their surveillance and diagnostic activities in sub-Saharan Africa. This study has therefore added to the body of knowledge and scientific data, the molecular evidence of the presence of the West African commonly found DENV-2 and DENV-3 serotypes in Ghana. This finding supports the co-circulation and concurrent infections by multiple DENV serotypes which has become a frequent occurrence in endemic countries [20,21].

Dengue serotype 2 has been dominant in West Africa [22] and this was given credence with the recent outbreaks in Burkina Faso and Senegal [17]. In an earlier hospital-based study conducted in a peri-urban area in Ghana, we sought to identify pathogens that cause febrile illnesses among children. Of the 700 enrolled, 2 were found to be acutely infected with DENV-2 when viral RNA was detected using molecular tools [23]. The dominance of Dengue 2 may be attributed to the prevalence of the strain of the transmissible vector in the sub-region. DENV serotypes and strains within the serotypes are known to vary in their ability to infect and disseminate in mosquitoes [24,25]. Dengue serotype 2 strains have been demonstrated to be better adapted and tend to infect the highly domesticated *Ae*. *aegypti* more efficiently [26]. It is thus considered the principal vector for DENV in urban areas whereas the species *Ae*. *albopictus* is recognized as an important vector in some rural areas [27].

The additional detection of DENV-3 and its relative proportion against DENV-2 in our study makes it a dual or multiple circulation of serotypes. This suggests possibility of concurrent serotype infections within the population. With the documented evidence that concurrent circulation by multiple Dengue serotypes influences clinical expression and accounts for the emergence of Dengue Hemorrhagic fevers (DHFs) [24], our finding presents a cause for public health concern in a country where hitherto hospital-based clinical and laboratory-based virologic surveillance system for DENV infection is inadequate. Since 1982 when the first case of concurrent infections with 2 DENV serotypes was reported in Puerto Rico [28], there have been documented reports of several concurrent infections in other countries [29, 30] and usually, though not in all cases, concurrent infections occur during epidemics or outbreaks. Our study finding of the co-circulating serotypes which also suggests hyperendemicity but without any apparent clinical or sub-clinical manifestation of DF or DHF may be partly due to the lack of hospital and laboratory-based surveillance at the time. It could also in part be attributed to the events at the time (during the peak of the EVD epidemic in West Africa) when the patients presented to the health facility were paid more attention because of the heightened alert for EVD suspected cases. Nevertheless, an absence of DHF notwithstanding hyperendemic co-circulating DENV transmission was also observed in a study in Haiti [31].

The rare clinical features observed with two of the four patients who tested positive for DF were in confused or disoriented state and unconsciousness. Although these features are usually not described to define a case of DF/DHF however, our observations are consistent with studies that have described unusual neurological manifestations of Dengue infection, including non-specific symptoms to encephalitis and, rarely, Guillain-Barré syndrome [32, 33]. Additionally, DENV-2 and DENV-3 are recurrently described as the cause of neurological sequelae [32]. It was worth noting that all the 4 positive patients had high grade fever and muscle/joint pains as main symptomatology in conformity with the WHO case definition [34] for DF. Further to that, 2 of the patients (IDs 50 and 73) had evidence of hemorrhagic manifestations, suggesting cases of DHF [33].

In our study analysis, we performed sequence phylogeny to confirm and establish the serotypes of the positive patient’s samples as documented reports in the sub-region on the circulation of different DENV serotypes are poor. Few publications however provide information on outbreaks and serological surveillance studies and reports that documented DENV infection in travellers. The results confirmed our serotypes as closely related to each other and clustered within DENV serotype 3 and in close homology with sequences from Senegal (KU509282) and India (FJ544564 and KU216208). Nonetheless, DENV-2 has been the main serotype reported to be circulating in West Africa [35] since the case of DENV was detected in Nigeria in 1964 [36]. In the West African countries of Côte d’Ivoire, Burkina Faso and Guinea, DENV-2 was detected from sylvatic cycles [37–39] and more closely in 2005, DENV-2 was identified in a traveller who returned from Ghana [40]. Despite this, DENV-3 has been detected severally in Africa [41, 42] with an instance where analysis suggested the importation of the virus from the Indian sub-continent [43]. Again, in our neighbouring country Côte d’Ivoire, DENV-3 was documented to have been co-circulating with Yellow fever [44]. These reports corroborate our finding of DENV-3 circulation in Ghana which has been noted to be consistent with evidence that this serotype is spreading in the sub-region [45].

In conclusion, we report the detection of 2 different serotypes of DENV circulating among patients suspected of EVD in Ghana. This makes for a public health interest on local DF burden and epidemiology and offers the platform to improve surveillance, laboratory testing capacity and clinical alertness in the health delivery system within the country and the sub-region.
